# Mediators of educational differences in dementia risk later in life: evidence from the HUNT study

**DOI:** 10.1186/s12889-025-22592-9

**Published:** 2025-04-10

**Authors:** Teferi Mekonnen, Vegard Skirbekk, Asta Kristine Håberg, Bo Engdahl, Ekaterina Zotcheva, Astanand Jugessur, Catherine Bowen, Geir Selbaek, Hans-Peter Kohler, Jennifer R. Harris, Sarah E. Tom, Steinar Krokstad, Trine Holt Edwin, Dana Kristjansson, Merete Ellingjord-Dale, Yaakov Stern, Bernt Bratsberg, Bjørn Heine Strand

**Affiliations:** 1 Department of Physical Health and Aging, Norwegian Institute of Public Health, Oslo, Norway; 2 Norwegian National Centre for Ageing and Health, Vestfold Hospital Trust, Tønsberg, Norway; 3 Centre for Fertility and Health, Norwegian Institute of Public Health, Oslo, Norway; 4 Department of Neuromedicine and Movement Science, Faculty of Medicine and Health Sciences, Norwegian University of Science and Technology, Trondheim, Norway; 5 Department of Global Public Health and Primary Care, University of Bergen, Bergen, Norway; 6 Independent Researcher, Vienna, Austria; 7 Department of Geriatric Medicine, Oslo University Hospital, Oslo, Norway; 8 Faculty of Medicine, University of Oslo, Oslo, Norway; 9 Population Aging Research Center, Department of Sociology, University of Pennsylvania, Philadelphia, PA USA; 10 Department of Neurology, Columbia University, Vagelos College of Physicians and Surgeons, New York, USA; 11 Department of Epidemiology, Mailman School of Public Health, Columbia University, New York, USA; 12 HUNT Research Centre, Department of Public Health and Nursing, Faculty of Medicine and Health Sciences, Norwegian University of Science and Technology, Trondheim, Norway; 13 Levanger Hospital, Nord-Trøndelag Hospital Trust, Levanger, Norway; 14 Department of Genetics and Bioinformatics, Norwegian Institute of Public Health, Oslo, Norway; 15 Ragnar Frisch Center for Economic Research, Oslo, Norway

**Keywords:** Dementia, Mediation, Educational differences

## Abstract

Despite a well-known inverse association between education and dementia risk, the mediating mechanisms are not well understood. We explored how lifestyle and health risk factors across the life-course mediate the relationship between education and dementia among adults aged 70 + years. We included 7,655 participants with dementia diagnoses and education information, using a historical cohort design linking prospective exposure data across the life course from the HUNT4 70 + Study with registry data from Statistics Norway and earlier HUNT surveys. We conducted causal mediation analysis to assess the mediating roles of occupational characteristics, lifestyle factors (smoking, physical inactivity), and health risk factors (obesity, hypertension, diabetes, hearing impairment, cardiovascular diseases, LDL cholesterol, depression, anxiety) assessed during early, middle, and late adulthood in the relationship between education and dementia in later life. Participants with lower education were more likely to have dementia with odds ratios of 1.99, 1.88, 1.83 for the model’s accounting exposure to mediators during early, middle, and late adulthood, respectively. These associations were partially mediated by the joint effect of health and lifestyle risk factors from early through late adulthood (mediated 11.55–19.50%). Health risk factors from early to late adulthood jointly mediated 6.85–13.06% of the effect of low education on dementia risk later in life. Additionally, lifestyle factors during middle and late adulthood jointly mediated 4.11–4.96% of the total effect of low education on dementia risk later in life. Educational differences in dementia risk can partly mediated by lifestyle and health factors across the life course. These findings suggest potential targets to address varying dementia risks linked to education levels.

**Supplementary Information:**

The online version contains supplementary material available at 10.1186/s12889-025-22592-9.

## Background

Dementia is a major global health problem [1, 2], with increasing prevalence [3, 4], and significant differences by level of education, affecting those in lower level of education compared to those with higher level of education [5, 6].

Educational attainment has been consistently linked to cognitive reserve and dementia risk [5–7], with lower educational levels associated with higher risk for Alzheimer’s disease and other dementias, although the causal relationship remains unclear [8, 9]. Understanding the mediating mechanisms by which education affects dementia risk using causal mediation analysis can help elucidate the relationship between education and dementia risk and ease causal interpretations of education’s effect on dementia risk.

Educational attainment is hypothesized to lower dementia risk by boosting cognitive reserve, i.e., the ability to maintain cognitive function despite presence of brain pathology, often attributed to factors like education, occupation, and lifestyle choices) [10], or by indirectly facilitating access to resources for health-promoting lifestyle behaviours such as better health literacy and healthier lifestyle options, both of which lower the risk or the consequences of chronic disease [11–14] and thereby lowering the risk of dementia. Studies have shown that individuals with a lower education are more likely to engage in unhealthy lifestyle factors such as smoking, physical inactivity, and excessive alcohol consumption. These individuals also have a higher risk of health risk factors such as obesity, hypertension, diabetes, and depression, compared to their higher-educated counterparts [12, 14–20]. However, few studies have explored which health risk factors, disease histories and occupational characteristics mediate the relationship between education and dementia risk late in life [21–23]. Previous studies indicate that educational differences in dementia risk is mediated by cardiovascular health (mediated 17%) [24]; vascular risk factors(mediated 11–25%) [21]; diabetes (mediated 0.004%) [23] and occupational characteristics (mediated 28%) [25].

Applying a life-course approach could improve the understanding of the link between education and dementia [26–28]. Identifying the factors that mediate the relationship between education and dementia risk at different life stages—early adulthood, middle adulthood, and late adulthood—can help pinpoint critical periods and target interventions to address educational disparities in dementia risk. In this regard, prior studies have mainly targeted exposure to risk factors during midlife [21, 24, 25]. In addition, sex differences in dementia risk [29–32] and related differences in risk factors, such as lifestyle and health related factors [33–35], have been reported, indicating a potential for sex-specific mediating pathways for educational differences in dementia risk. Nevertheless, research on sex-specific mediating pathways for the educational differences in dementia risk is lacking. In this study, we aimed to assess the mediating roles of lifestyle, health risk factors and occupational characteristics across the life course in the relationship between education and dementia. In addition, we explored sex-specific mediating pathways.

## Methods

### Study population and design

**Fig. 1:**
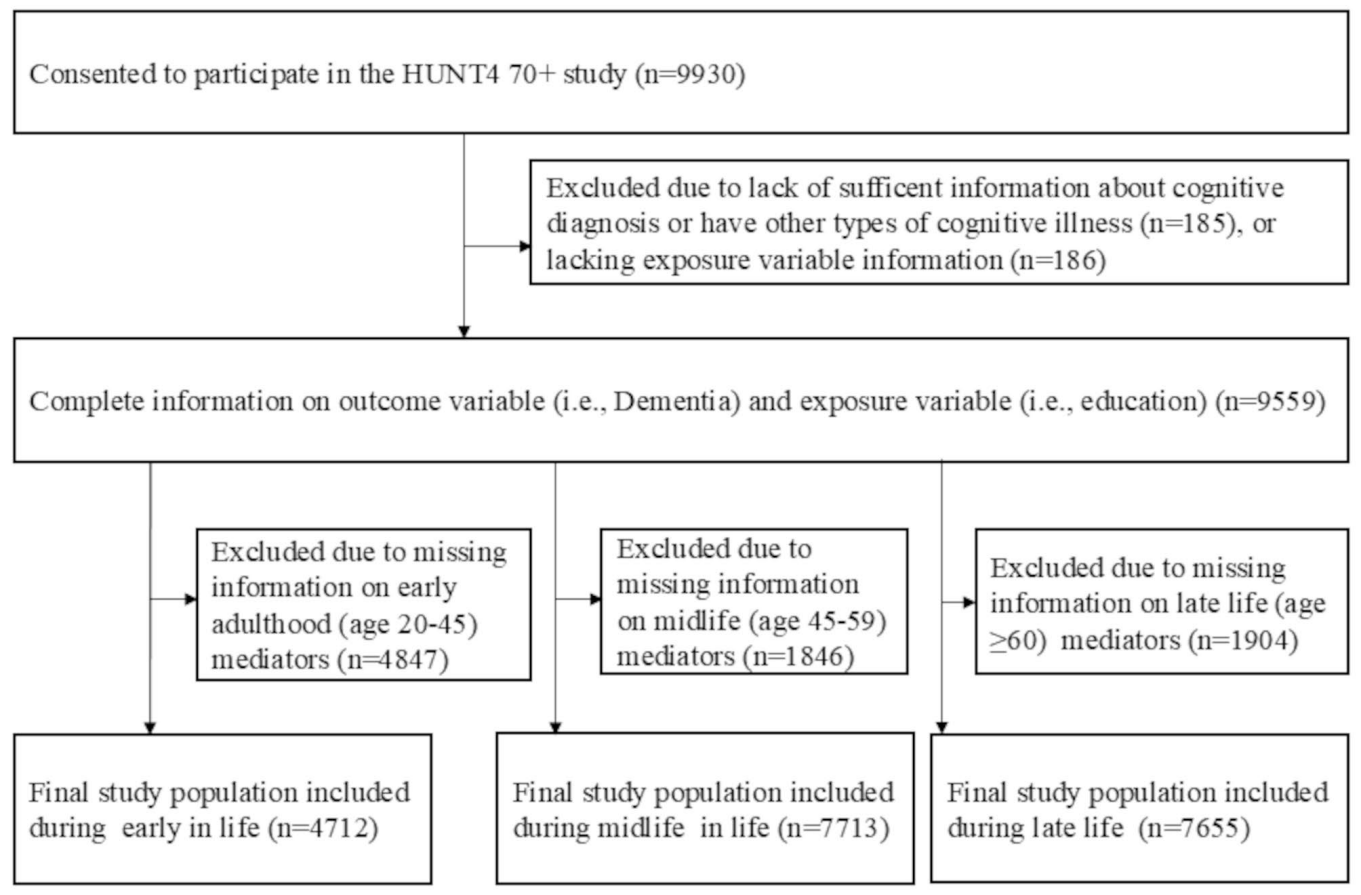
Overview of the sampling scheme: the HUNT Study, Norway

We used a historical cohort design, linking older adults aged 70 + years who underwent clinical cognitive assessment in the HUNT4 70 + study, conducted from 2017 to 2019, with administrative prospective data from Statistics Norway and previous HUNT surveys: HUNT1 (1984–1986), HUNT2 (1995–1997), and HUNT3 (2006–2008) (39). All adult inhabitants aged 70 years and above, residing in the former Nord-Trøndelag County of Norway, were invited to participate in in the HUNT4 survey. HUNT1–HUNT4 [ [36, 37]. Of those who consented, 96% participated in at least one of the HUNT study surveys, 90.5% participated in at least two of the HUNT1 to HUNT3 surveys, and 77% participated in all previous surveys (HUNT1–HUNT3 [36, 37]. Among the eligible participants (*n* = 9930) in the HUNT4 70 + study, who were linked to dementia diagnoses and previous HUNT study waves (i.e., HUNT1, HUNT2, HUNT3) as well as registries, we excluded individuals who either lacked information about their cognitive diagnosis or had cognitive disorders other than mild cognitive impairment or dementia (*n* = 185). Further exclusions were made for those with missing information on educational status (*n* = 186), yielding a total of 9559 participants with a complete information on education and dementia diagnosis status. Study participants were defined at three stages of adult life: early adulthood (20–44 years), middle adulthood (45–59 years), and late adulthood (≥ 60 years) based on the age at which mediators were assessed. Additional exclusions were made if there was missing information for the mediators at each stage of life, yielding final eligible participants during early adulthood (*n* = 4712), middle adulthood (*n* = 7713), and late adulthood (*n* = 7655) (Fig. 1).

### Outcome assessment

The primary outcome variable was dementia status, categorized as either ‘yes’ (presence of dementia) or ‘no’ (absence of dementia). All participants underwent a detailed evaluation by trained health personnel, which assessed cognitive function, daily-life abilities, neuropsychiatric symptoms, and subjective cognitive decline. Additionally, interviews were conducted with next-of-kin to collect further information. Dementia classification was based on DSM-5 criteria and conducted post-hoc by two independent physicians chosen from a group of nine specialists in geriatrics, old-age psychiatry, or neurology. In the case of disagreement, a third specialist was conferred. Participants were assigned to one of five categories: (0) no cognitive impairment, (1) amnestic mild cognitive impairment, (2) non-amnestic mild cognitive impairment, (3) dementia, and (4) either insufficient cognitive diagnosis information or other cognitive disorders (Gjøra, Strand et al. 2021). For this analysis, a binary variable was created: individuals in category 3 were classified as ‘having dementia,’ while those in categories 0, 1, and 2 were classified as ‘not having dementia.’ Participants in category 4 were excluded from the study.

### Exposure assessment

Educational status information was obtained from Statistics Norway, reported in 1970 (FOB1960-1980 survey), when study participants were, on average, aged 30 ± 6.5 years. The level of education was reported in year completed, ranging from 0 to 19 years. Educational status was categorized as “lower secondary education” for participants who had completed up to 9 years of education and “upper secondary education and above” for those who had completed 10 or more years of education, hereafter denoted low and high education, respectively. Evidence suggests that attaining more than 10 years of education is associated with a reduced risk of dementia [38, 39].

### Assessment of potential mediators

**Fig. 2:**
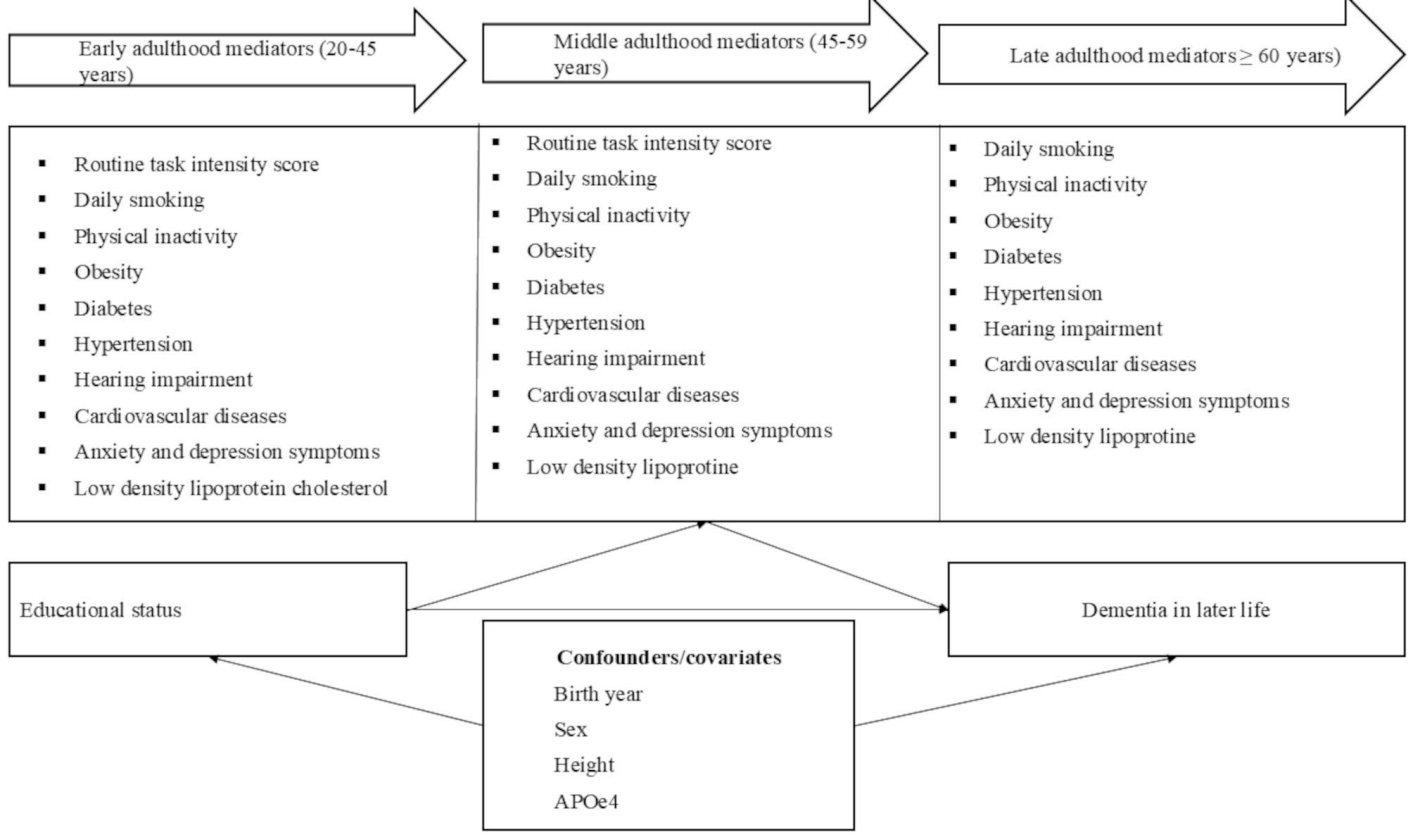
Directed acyclic graph linking educational status to dementia in later life

Selection of potential mediators was inspired by the Lancet commission on dementia prevention, intervention and care 2024 [40], and availability of potential mediators in our dataset (Fig. 2). We categorized the mediators based on exposure at different life stages: early adulthood, middle adulthood, and later adulthood. Supplementary Table 1 provides details on how the mediators were assessed and defined, and the sources of the data.

The mediators considered in our analysis were occupational characteristics assessed using routine task intensity score [41]. We used data from Statistics Norway’s administrative registries through a unique personal identification number provided to all registered residents in Norway. We imputed the ISCO-88 occupational codes using a crosswalk approach that combined a four-digit industry of employment code and a four-digit educational code. The O*NET (2003 version)17 was used to establish the degree to which each occupation involved performance of routine manual, routine cognitive, nonroutine analytical, and nonroutine interpersonal tasks. The routine task intensity (RTI) score, representing occupational cognitive demands, was calculated and categorized into quartiles using standardized scores (1st, 2nd, 3rd, and 4th quartiles), with an additional category for individuals who were unemployed or had missing employment data. A lower RTI score reflects a lower degree of routine work, suggesting greater cognitive demands in the occupation. Health risk factors including daily smoking (never daily smoked, daily smoked before, current daily smoker), physical inactivity (yes vs. no), obesity (yes vs. no), hypertension (yes vs. no), diabetes (yes vs. no), hearing impairment (yes vs. no), cardiovascular diseases (yes vs. no), LDL, and anxiety and depression symptoms assessed using the 14-item Hospital Anxiety and Depression Scale (HADS). A license to use the Hospital Anxiety and Depression Scale was obtained, and proof of this permission has been provided. To simplify the interpretation of our findings, we grouped mediators into occupational characteristics (i.e., routine task intensity score), lifestyle factors (i.e., daily smoking, physical inactivity), and health risk factors (i.e., obesity, hypertension, diabetes, hearing impairment, cardiovascular diseases, LDL, and HADS).

### Potential covariates/confounders

The potential confounders included in our analysis were sex (male vs. female), APOe4 (has no APOe4, one APOe4, or two APOe4), height (in centimeters) and birth year (born 1940 or before vs. born after 1940).

### Statistical analysis

Using the mma mediation R package [42], we investigated whether potential mediators assessed during early, middle, and late adulthood mediated the relationship between education and the risk of dementia. Separate mediation models, adjusted for confounders were fitted to assess both the joint and independent mediating roles of exposure to potential mediators during early, middle, and late adulthood. In addition, we examined sex-specific mediating pathways of educational differences in dementia risk. The direct, indirect, and total effects with 95% confidence intervals (CI) were estimated using 200 bootstrap replication and the proportion mediated with 95% CI was presented. The direct effect represents the remaining effect of education (low vs. high) on dementia if we eliminate the effect the pathway from educational status to the mediators, given a set of covariates or confounders. The total effect of education on dementia refers to the overall change in the outcome if the exposure status were altered from unexposed (low education) to exposed (high education), given a set of covariates or confounders. The indirect effect captures the difference between the total effect and the direct effect of education on dementia risk, isolating the portion of the effect that operates through mediators [42]. The percentage mediated effect was estimated by dividing the indirect effect of mediators of interest to the total effect of exposure variable on the outcome variable [43]. STATA 17/SE and R 4.1.2 were used for data cleaning and mediation analyses, respectively.

## Results

### Participants characteristics

Table 1 describes characteristics of study participants during early, middle and late adulthood. Participants with lower education showed higher proportion of dementia compared to those higher levels of education across all life stages considered in our study. Participants with lower education more often reported an unfavorable lifestyle and health risk factors across all life stages considered in this study. Regarding occupational characteristics, study participants were more likely to be in the higher quartiles of routine task intensity scores or to have no occupational information/not working, compared to those with high education during early and middle adulthood. This was observed for participants considered for exposure to potential mediators during early adulthood, middle adulthood and late adulthood (Table 1).

**Table 1:** Characteristics of study participants by level of education during early, middle and late adulthood

	Early adulthood			Middle adulthood			Late adulthood		
	Total	Educational status		Total	Educational status		Total	Educational status	
		High	Low		High	Low		High	Low
	(*N* = 4712)	(*N* = 2239)	(*N* = 2473)	(*N* = 7713)	(*N* = 3281)	(*N* = 4432)	(*N* = 7655)	(*N* = 3191)	(*N* = 4464)
	*N* (%)	*N* (%)	*N* (%)	*N* (%)	*N* (%)	*N* (%)	*N* (%)	*N* (%)	*N* (%)
Dementia status									
*No*	4398 (93)	2138 (95)	2260 (91)	6685 (87)	3001 (91)	3684 (83)	6496 (85)	2885 (90)	3611 (81)
*Yes*	314 (7)	101 (5)	213 (9%)	1028 (13)	280 (9)	748 (17)	1159 (15)	306 (10)	853 (19)
Sex									
*Male*	2528 (54)	1092 (49)	1436 (58)	4278 (55)	1536 (47)	2742 (62)	4268 (56)	1478 (46)	2790 (62.)
*Female*	2184 (46)	1147 (51)	1037 (42)	3435 (45)	1745 (53)	1690 (38)	3387 (44)	1713 (54)	1674 (38)
ApoE4									
*No e4*	3257 (69)	1543 (69)	1714 (69)	5390 (70)	2276 (69)	3114 (70)	5370 (70)	2220 (70)	3150 (71)
*One e4*	1307 (28)	626 (28)	681 (28)	2119 (27)	918 (28)	1201 (27)	2092 (27)	892 (28)	1200 (27)
*Two e4*	148 (3)	70 (3)	78 (3)	204 (3)	87 (3)	117 (3)	193 (3)	79 (2)	114 (2)
Year of birth									
*Born 1940 or before*	389 (8)	157 (7)	232 (9)	3385 (44)	1195 (36)	2190 (49)	3618 (47)	1250 (39)	2368 (53)
*Born after 1940*	4323 (92)	2082 (93)	2241 (91)	4328 (56)	2086 (64)	2242 (51)	4037 (53)	1941 (61)	2096 (47)
Height									
*Mean (SD)*	170 ± 9	170 ± 9	170 ± 9	170 ± 9	170 ± 9	170 ± 9	170 ± 9	172 ± 9	169 ± 9
Hypertension									
*No*	3489 (74)	1698 (76)	1791 (72)	5909 (77)	2643 (81)	3266 (74)	6288 (82)	2720 (85)	3568 (80)
*Yes*	1223 (26)	541 (24)	682 (28)	1804 (23)	638 (19)	1166 (26)	1367 (18)	471 (15)	896 (20)
Daily smoking									
*Never smoked*	1998 (42)	1078 (48)	920 (37)	3639 (47)	1668 (51)	1971 (44)	3732 (49)	1663 (52)	2069 (46)
*Daily smoked before*	1401 (30)	653 (29)	748 (30)	2826 (37)	1202 (37)	1624 (37)	3114 (41)	1270 (40)	1844 (41)
*Daily smoker*	1313 (28)	508 (23)	805 (33)	1248 (16)	411 (13)	837 (19)	809 (11)	258 (8)	551 (12)
Obesity									
*No*	4461 (95)	2153 (96)	2308 (93)	7248 (94)	3140 (96)	4108 (93)	7418 (97)	3120 (98)	4298 (96)
*Yes*	251 (5)	86 (4)	165 (7)	465 (6)	141 (4)	324 (7)	237 (3)	71 (2)	166 (3)
Physical inactivity									
*No*	2603 (55)	1375 (61)	1228 (50)	5464 (71)	2575 (78)	2889 (65)	6022 (79)	2696 (85)	3326 (74)
*Yes*	2109 (45)	864 (39)	1245 (50)	2249 (29)	706 (22)	1543 (35)	1633 (21)	495 (15)	1138 (25)
Diabetes									
*No*	4612 (98)	2197 (98)	2415 (98)	7515 (97)	3195 (97)	4320 (97)	7570 (99)	3153 (99)	4417 (99)
*Yes*	100 (2)	42 (2)	58 (2)	198 (3)	86 (3)	112 (3)	85 (1)	38 (1)	47 (1)
Hearing impairment									
*No*	4576 (97)	2186 (98)	2390 (97)	7476 (97)	3205 (98)	4271 (96)	7477 (98)	3141 (98)	4336 (97)
*Yes*	136 (3)	53 (2)	83 (3)	237 (3)	76 (2)	161 (4)	178 (2.3)	50 (1)	128 (3)
Cardiovascular diseases									
*No*	4583 (97)	2197 (98)	2386 (96)	7345 (95)	3169 (97)	4176 (94)	7245 (95)	3068 (96)	4177 (94)
*Yes*	129 (3)	42 (2)	87 (4)	368 (5)	112 (3)	256 (6 is)	410 (5)	123 (3)	287 (6)
RTI score									
*1st quartile*	1293 (27)	895 (40)	398 (16)	1548 (20)	1180 (36)	368 (8)	-	-	-
*2nd quartile*	866 (18)	573 (26)	293 (12)	1951 (25)	841 (26)	1110 (25)			
*3rd quartile*	966 (21)	337 (15)	629 (25)	1154 (15)	383 (12)	771 (17)	-	-	-
*4th quartile*	1030 (22)	257 (11)	773 (31)	1553 (20)	420 (13)	1133 (26)			
Missing/not working	557 (12)	177 (8)	380 (15)	1507 (20)	457 (14)	1050 (24)	-	-	-
LDL level									
*Mean (± SD)*	150 ± 33	150 ± 32	150 ± 33	150 ± 33	150 ± 32	150 ± 34	150 ± 33.6	146 ± 32	153 ± 34
Depression and anxiety symptoms									
*Mean (SD)*	8 ± 5	7 ± 5	8 ± 5	8 ± 5	7 ± 5	8 ± 5	8 ± 5	7 ± 5	8 ± 5

### Total effect of education and later life dementia risk

**Fig. 3:**
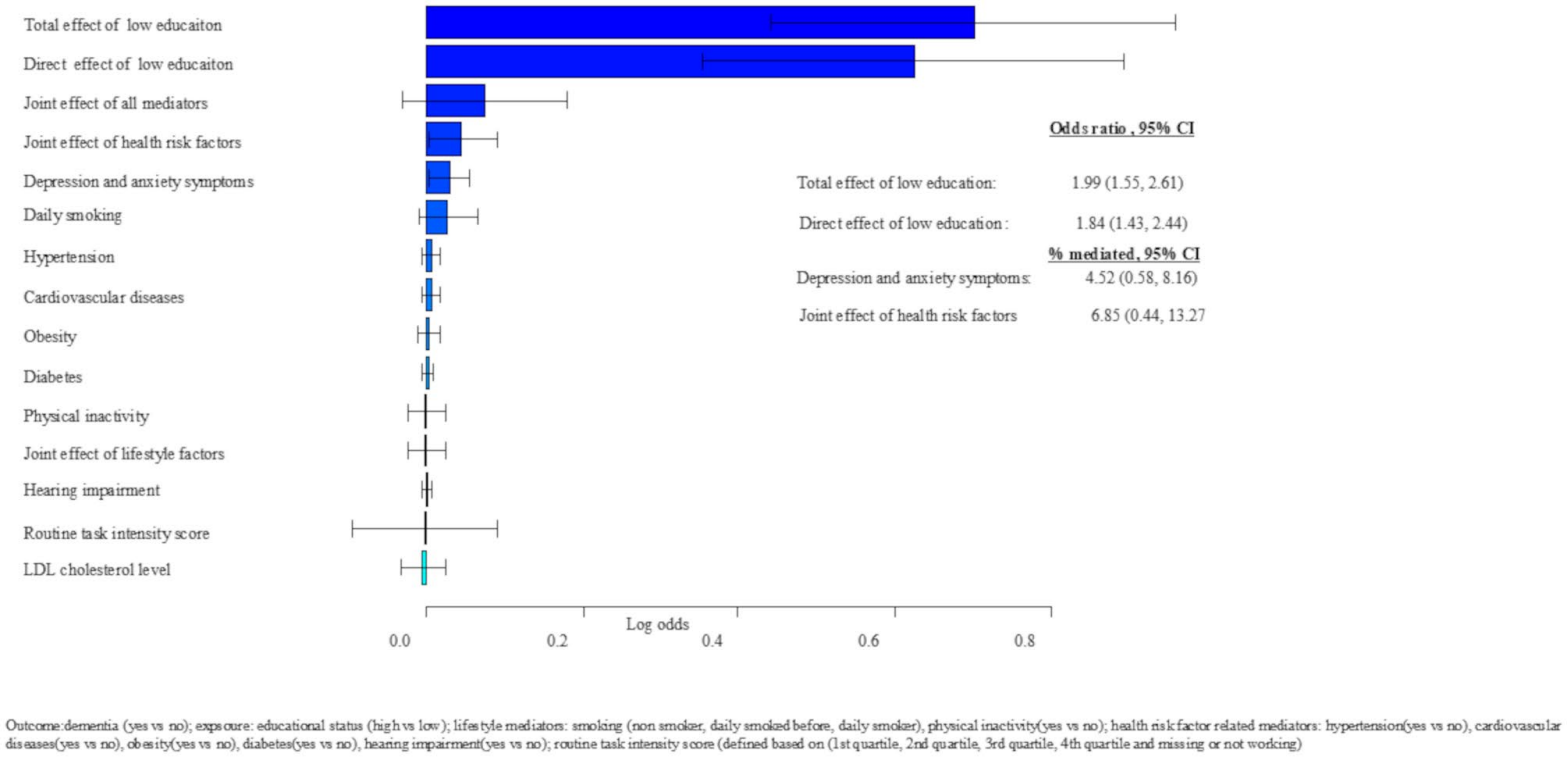
Early adulthood mediators of educational difference in dementia risk in later life, adjusted for sex, APOE4, height, and birth year

**Fig. 4:**
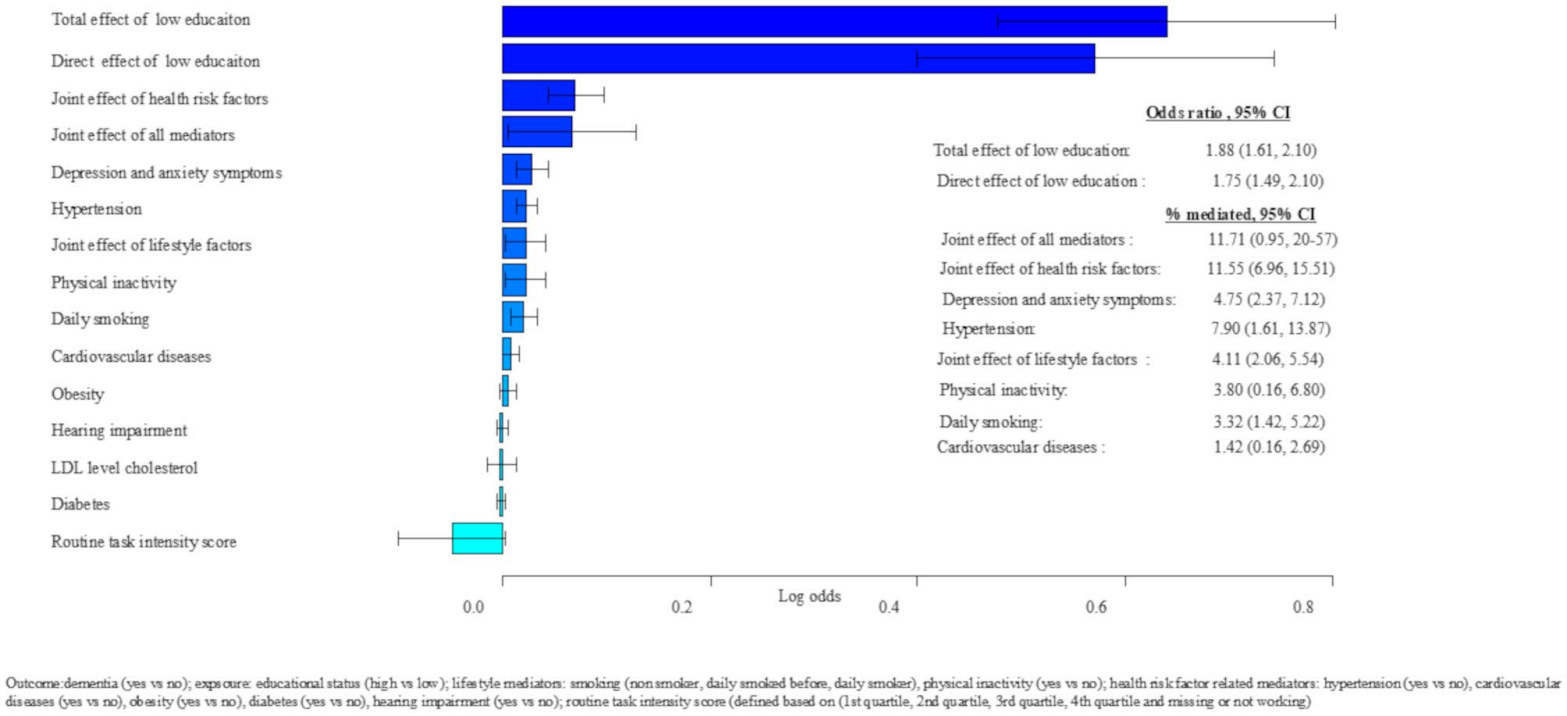
Middle adulthood mediators of educational difference in dementia risk in later life, adjusted for sex, APOE4, height, and birth year

**Fig. 5:**
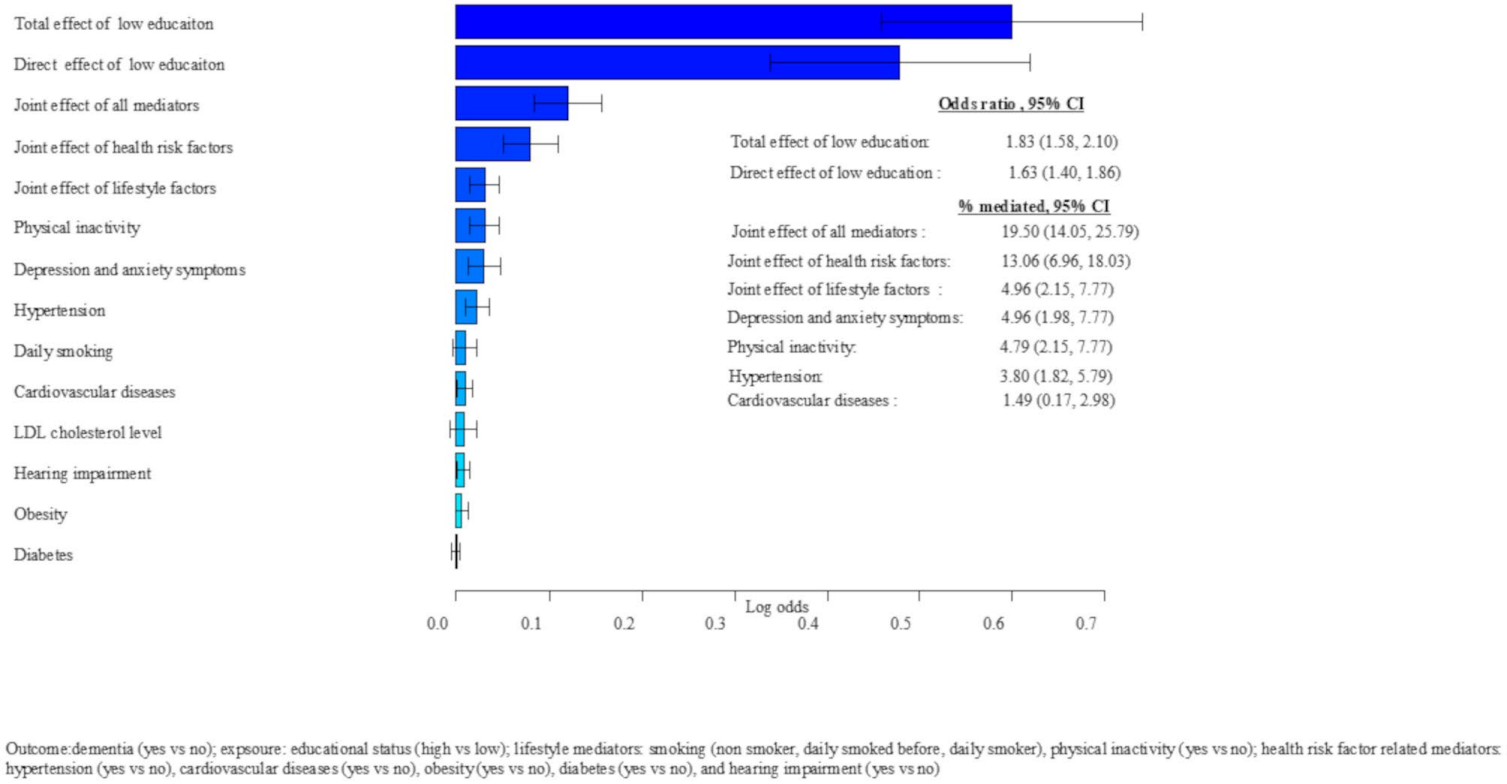
Late adulthood mediators of educational difference in dementia risk in later life, adjusted for sex, APOE4, height, and birth year

Adults with low education had higher odds of dementia compared to those with high education for the model during early adulthood (total effect [TE]: 1.99, 95% CI: 1.55, 2.61), middle adulthood (TE: 1.88, 95% CI: 1.61, 2.10), and late adulthood (TE: 1.83, 95% CI: 1.58, 2.10) (Figs. 3, 4 and 5).

### Direct effect of education and later life dementia risk

The direct effect of education (low vs. high) on dementia after accounting for the mediators assessed during early, middle, and late adulthood, a statistically significant direct effect of education was odds ratio (OR): 1.84 (95% CI 1.43, 2.44) during early adulthood, OR = 1.75 (95% CI 1.49, 2.10) during middle adulthood, and OR = 1.63 (95% CI 1.40, 1.86) during late adulthood. This indicates that the mediators considered in our analyses had a partial mediation effect (Figs. 3, 4 and 5).

### Indirect effect of education on dementia risk later in life: the mediating role of early adulthood risk factors

Among all the mediators considered during early adulthood, health risk factors jointly mediated 6.85% of the effect of low education on dementia risk later in life, while anxiety and depression symptoms independently mediated 4.52% of the total effect of low education on dementia risk later in life (Fig. 3).

### Indirect effect of education on later life dementia risk: the mediating role of middle adulthood risk factors

All mediators assessed during middle adulthood jointly mediated 11.71% of the total effect of low education on dementia risk later in life. Health risk factors and lifestyle factors assessed during middle adulthood jointly mediated 11.55% and 4.11% of the total effect of low education on dementia risk later in life, respectively. Among the middle adulthood mediators considered in our analysis, anxiety and depression symptoms, hypertension, physical inactivity, daily smoking, and cardiovascular diseases showed independent mediating effects, explaining 4.75%, 3.9%, 7.90%, 3.32%, and 1.42% of the total effect of low education on dementia risk (Fig. 4).

### Indirect effect of education on dementia risk in later life: the mediating role of later adulthood risk factors

Mediators assessed during late adulthood (excluding occupational characteristics) jointly mediated 19.50% of the total effect of low education on dementia risk later in life. Late life health risk factors and lifestyle factors considered in our study mediated 13.06% and 4.96% of the effect of low education on dementia risk later in life, respectively. Anxiety and depression symptoms, physical inactivity, hypertension, cardiovascular diseases during late adulthood showed independent mediating effects, mediating 4.96%, 4.79%, 3.80% and 1.49% of the total effect of low education on dementia risk later in life, respectively (Fig. 5).

### Sex-specific mediating paths for educational differences in later life dementia risk

None of the potential mediators in early adulthood, except anxiety and depression symptoms among females (mediating 7.85%), significantly mediated the relationship between education and dementia risk in the sex-stratified analysis (Supplementary Figs. 1). Health risk factors during middle adulthood mediated 7.85% of the effect in females and 16.61% in males, while all middle adulthood factors together mediated 25.00% in males (Supplementary Figs. 2). Late adulthood health factors mediated 10.40% in females and 19.48% in males, and late-life lifestyle factors mediated 2.64% in females and 6.31% in males. All late-life factors combined mediated 17.28% in females and 30.06% in males (Supplementary Figs. 3).

### Sensitivity analysis of all life stages together

Our analysis results, accounting for mediators at all life stages together, showed that health risk factors altogether mediated 21.55% of the effect of education on later-life dementia risk, while all mediators combined accounted for 15.90% of the effect. Among individual mediators, hearing impairment (8.48%), depression and anxiety symptoms (4.42%), hypertension (4.24%), and cardiovascular diseases (1.77%) explained the effect of education on later-life dementia risk Supplementary Figs. 4).

## Discussion

In this large Norwegian population-based historical cohort study, we found that the increased dementia risk associated with low education was partially mediated by various health and lifestyle factors assessed from early to late adulthood. In total, these factors explained less than one-fifth of the educational differences in dementia risk later in life. Moreover, 7–13% of the relationship between education and dementia risk in late life was mediated by health risk factors during early, middle, and late adulthood, and about 5% was mediated by lifestyle factors during middle and late adulthood. Anxiety and depression symptoms across all adult life stages, physical inactivity, hypertension, cardiovascular diseases from middle and late adulthood, and smoking during middle adulthood were factors that showed an independent mediating effects for the relationship between low education and dementia risk in later life. Our findings also highlighted relatively stronger mediated effect for of health risk factors and all mediators combined for the effect of low education on dementia risk among males compared to females.

The increased risk of dementia later in life among individuals with lower education, compared to those with higher education, in our study is consistent with meta-analyses and systematic reviews, which report a higher risk of dementia among individuals with a lower level of education compared to their highly educated counterparts [5–7]. The mechanisms for the inverse relationship between educational attainment and dementia risk in later life could possibly be due to the influence of education on lifestyle and health risk factors. Existing studies in this regard have shown that the relationship between education and dementia risk can be explained by factors such as systolic blood pressure, fasting blood glucose, smoking, body mass index, adherence to diet, and physical inactivity [21]. These factors jointly mediated 11–24% of the effect of education on dementia risk using different education categories, which is comparable with our findings, particularly regarding the mediating effect of middle adulthood mediators for the high school without graduation groups. However, the mediating effect of midlife mediators in our study was lower than that of a study which used a composite cardiovascular risk score, computed from smoking, physical activity, healthy diet, body mass index, total cholesterol, blood pressure, and fasting plasma glucose. This composite score mediated 17% of the educational differences in dementia risk later in life [24], where the discrepancies could possibly be due to the way the mediators were defined, as they used a cardiovascular health score.

Our findings indicated that lifestyle factors (i.e., daily smoking and physical inactivity assessed during middle and late adulthood) jointly mediated up to 5% of the relationship between low education and dementia risk in later life. Prior studies have provided evidence of a strong relationship between smoking during midlife [44] and physical inactivity during middle and late life [45, 46] with dementia risk, as well as differences in these risk factors by level of education [47, 48]. The findings regarding the mediating role of these factors in our study underscore the importance of lifestyle choices, such as avoiding smoking and being physically active during mid and late adulthood, to prevent later life dementia risk among individuals with lower educational attainment.

Among the health risk factors considered in our study, anxiety and depressive symptoms present throughout all life stages during adulthood, along with hypertension and cardiovascular diseases from middle adulthood onwards showed independent mediating effect for relationship between low education dementia risk. Our finding for the effect of hypertension during middle adulthood was particularly higher compared to a study conducted in the US (explained 2–5%) the effect of high school education without graduation on dementia risk [21]. The discrepancies could partly be explained by the operationalization of educational status and the definition of hypertension. In our study, we defined hypertension and used a binary exposure, whereas the US study used systolic blood pressure on a continuous scale and defined educational status differently.

Although there is an emphasis on the importance of identifying risk factors from a life course perspective to pinpoint intervention entry points for addressing health inequalities, including dementia [27, 49], existing research has primarily examined midlife mediators of educational differences in dementia risk in later life. Less attention has been paid to factors from early adulthood and factors late adulthood period. Our findings contribute to the existing body of evidence by quantifying the mediating role of specific risk factors throughout the life course and underscored the importance of addressing anxiety and depression symptoms across all life stages, hypertension and cardiovascular diseases, and lifestyle choices such as midlife smoking and physical inactivity during late adulthood. By targeting these risk factors, it may be possible to delay or prevent later life dementia risk, particularly among those with lower levels of education.

The fact that middle and late adulthood mediators have a more pronounced mediating effect for the association between education and dementia risk in later life among males could partly be due to differences in health risk factors and lifestyle factors between males and females. For example, existing studies have shown a stronger association between lifestyle behaviors, such as alcohol intake [50], and being at a higher risk for certain health conditions, such as heart disease and stroke, in men than in women [32, 51], which could explain the stronger mediating effect among male than female adults.

Even considering a range of mediating factors in our and other studies, a substantial proportion of the association of education dementia risk in later life remains unexplained [21, 24], suggesting that there might be other mechanisms which explain the relationship between education and dementia risk later in life. This may partly be explained by cognitive reserve which benefits individuals with higher education by enabling them to better compensate for the brain changes associated with dementia, delaying the onset of symptoms [6]. Given that dementia can be caused by a range of risk factors interacting with each other [40], further studies including more potential mediators are recommended to understand the mediating mechanism of how low education contributes dementia risk in later life.

From a policy perspective, the substantial direct effect of education suggests that investments in early-life education may yield long-term cognitive benefits that extend beyond what can be attributed to a range of health risk factors and lifestyle-related influences in adulthood. Policies aimed at improving access to quality education, particularly for disadvantaged populations, could enhance cognitive resilience and potentially reduce disparities in age-related cognitive decline and dementia risk. While promoting healthy behaviors and preventing chronic disease remains essential, our findings suggest that such interventions may not fully compensate for educational disparities. This underscores the need for a dual approach—expanding educational opportunities in early life while also implementing targeted lifestyle and chronic disease interventions throughout adulthood.

### Strength and limitations

The strengths of this study include the large sample size, (population-based sampling) standardized approach to dementia diagnoses, measurements of health risk factors and lifestyle factors across the adult life course, use of high-quality registry data for defining educational status, and the integration of a life-course approach with mediation analysis to decompose the total effect of education and dementia risk into direct and indirect effects. However, this study also has its limitations. One major limitation is that mediation analysis relies on the assumption of no unmeasured confounding [52–54]. However, the outcome variable, dementia, and potential mediators, such as lifestyle behaviors and markers of chronic diseases, may be influenced by various unmeasured factors. Assuming no confounders in the mediator-outcome relationships is challenging, as many residual variables are either inaccessible or unknown. One possible confounding factor is inherent intellectual ability, which is possibly linked to cognitive activity and affects educational achievement of an individual. Despite our efforts to minimize and account for confounders, eliminating all potential confounders is difficult in practice. In this study, using dementia diagnosis information conducted by medical doctors following DSM-5 criteria, and a thorough clinical examination of cognitive function, activities of daily living, neuropsychiatric symptoms, and subjective cognitive decline, as well as interviews with next-of-kin [55], we defined dementia status as either “having dementia” vs. “having no dementia or living with mild cognitive impairment”. Placing individuals with mild cognitive impairment into the same category as cognitively healthy individuals may have biased our results toward the null. Further, using only two educational groups (lower vs. high) means that we potentially missed out on important educational variations in dementia risk. However, to ensure sufficient power and facilitate the mediation analyses, dichotomization of educational attainment was necessary in our study. Our study exhibits a healthy selection bias, as participation in HUNT surveys is influenced by survival, socioeconomic status, and the absence of chronic diseases [56]. Non-participants in HUNT surveys tend to have unhealthy lifestyle behaviors, such as smoking and physical inactivity, and markers of chronic diseases like diabetes, mental distress, and cardiovascular diseases [56]. Such selection could have an effect in our mediation parameters that could potentially underestimate the mediated effect. In addition, given that our outcome depends on survival at age 70 years and evidence of survival bias [56], ignoring competing risk, as we have done, might introduce bias [57]. The indirect effect estimates for the factors such as lifestyle behaviors and chronic diseases might be underestimated [58, 59], if there is competing risk, as has been previously reported in smoking [57]. Mediators such as physical activity and smoking status were self-reported, which may be subjected to information bias. This could weaken the associations between mediators and outcomes. In such cases, the mediated effect might be biased towards the null.

## Conclusions

Educational differences in dementia risk later in life can be partly explained by modifiable risk factors such as lifestyle and health risk factors across life course. Anxiety and depression symptoms across all adult life stages, physical inactivity, hypertension, cardiovascular diseases from middle and late adulthood, and smoking during middle adulthood plays significant roles to educational differences in dementia risk in life. If truly causal, the findings could indicate potential targets for interventions to tackle educational differences in dementia risk among older people.

## Electronic supplementary material

Below is the link to the electronic supplementary material.

Supplementary Material 1

Supplementary Material 2

## Data Availability

The data used in the current study are available after approval by the Regional Committee for Medical and Health Research Ethics and HUNT’s Data Access Committee.

## Abbreviations

HUNT: Helseundersøkelsene i Trøndelag
FOB: Folke- og boligtellingen
O*NET: Occupational Information Network
RTI: Routine task intensity
TE: Total effect
OR: Odds Ratio
HADS: Hospital anxiety depression score

## References

[1] Vos T, Barber RM, Bell B, Bertozzi-Villa A, Biryukov S, Bolliger I, Charlson F, Davis A, Degenhardt L, Dicker D. Global, regional, and National incidence, prevalence, and years lived with disability for 301 acute and chronic diseases and injuries in 188 countries, 1990–2013: a systematic analysis for the global burden of disease study 2013. Lancet. 2015;386(9995):743–800. 26063472 10.1016/S0140-6736(15)60692-4 PMC4561509

[2] Serge Gauthier PR-N, José A, Morais CW. World alzheimer report 2021: journey through the diagnosis of dementia. In. London, England: Alzheimer’s Disease International; 2021.

[3] Nichols E, Steinmetz JD, Vollset SE, Fukutaki K, Chalek J, Abd-Allah F, Abdoli A, Abualhasan A, Abu-Gharbieh E, Akram TT. Estimation of the global prevalence of dementia in 2019 and forecasted prevalence in 2050: an analysis for the global burden of disease study 2019. Lancet Public Health. 2022;7(2):e105–25. 34998485 10.1016/S2468-2667(21)00249-8 PMC8810394

[4] Cao Q, Tan C-C, Xu W, Hu H, Cao X-P, Dong Q, Tan L, Yu J-T. The prevalence of dementia: a systematic review and meta-analysis. J Alzheimers Dis. 2020;73(3):1157–66. 31884487 10.3233/JAD-191092

[5] Wang A-Y, Hu H-Y, Ou Y-N, Wang Z-T, Ma Y-H, Tan L, Yu J-T. Socioeconomic status and risks of cognitive impairment and dementia: a systematic review and meta-analysis of 39 prospective studies. J Prev Alzheimer’s Disease. 2023;10(1):83–94. 36641612 10.14283/jpad.2022.81

[6] Meng X, D’arcy C. Education and dementia in the context of the cognitive reserve hypothesis: a systematic review with meta-analyses and qualitative analyses. PLoS ONE. 2012;7(6):e38268. 22675535 10.1371/journal.pone.0038268 PMC3366926

[7] Sharp ES, Gatz M. The relationship between education and dementia an updated systematic review. Alzheimer Dis Assoc Disord. 2011;25(4):289. 21750453 10.1097/WAD.0b013e318211c83c PMC3193875

[8] Seblova D, Fischer M, Fors S, Johnell K, Karlsson M, Nilsson T, Svensson AC, Lövdén M, Lager A. Does prolonged education causally affect dementia risk when adult socioeconomic status is not altered? A Swedish natural experiment in 1.3 million individuals. Am J Epidemiol. 2021;190(5):817–26. 33226079 10.1093/aje/kwaa255

[9] Amin V, Behrman J, Fletcher JM, Flores CA, Flores-Lagunes A, Kohler H-P. Does schooling improve cognitive abilities at older ages: causal evidence from nonparametric bounds. 2022. 10.1215/00703370-11865131 PMC12702574 40152756

[10] Stern Y, Gurland B, Tatemichi TK, Tang MX, Wilder D, Mayeux R. Influence of education and occupation on the incidence of Alzheimer’s disease. JAMA. 1994;271(13):1004–10. 8139057

[11] Andersen R, Van de Werfhorst HG. Education and occupational status in 14 countries: the role of educational institutions and labour market coordination. Br J Sociol. 2010;61(2):336–55. 20579057 10.1111/j.1468-4446.2010.01315.x

[12] Clouston SA, Richards M, Cadar D, Hofer SM. Educational inequalities in health behaviors at midlife: is there a role for early-life cognition? J Health Soc Behav. 2015;56(3):323–40. 26315501 10.1177/0022146515594188 PMC4678035

[13] Graff-Iversen S, Ariansen I, Næss Ø, Selmer RM, Strand BH. Educational inequalities in midlife risk factors for non-communicable diseases in two Norwegian counties 1974–2002. Scand J Public Health. 2019;47(7):705–12. 30080116 10.1177/1403494818789325

[14] Nagel G, Peter R, Braig S, Hermann S, Rohrmann S, Linseisen J. The impact of education on risk factors and the occurrence of Multimorbidity in the EPIC-Heidelberg cohort. BMC Public Health. 2008;8:1–10. 19014444 10.1186/1471-2458-8-384 PMC2614432

[15] Park C, Kang C. Does education induce healthy lifestyle? J Health Econ. 2008;27(6):1516–31. 18963121 10.1016/j.jhealeco.2008.07.005

[16] Koning P, Webbink D, Martin NG. The effect of education on smoking behavior: new evidence from smoking durations of a sample of twins. Empirical Economics. 2015;48:1479–97.

[17] Droomers M, Schrijvers CT, Stronks K, van de Mheen D, Mackenbach JP. Educational differences in excessive alcohol consumption: the role of psychosocial and material stressors. Prev Med. 1999;29(1):1–10. 10419792 10.1006/pmed.1999.0496

[18] Choi AI, Weekley CC, Chen S-C, Li S, Tamura MK, Norris KC, Shlipak MG. Association of educational attainment with chronic disease and mortality: the kidney early evaluation program (KEEP). Am J Kidney Dis. 2011;58(2):228–34. 21601328 10.1053/j.ajkd.2011.02.388 PMC3144262

[19] Dupre ME. Educational differences in health risks and illness over the life course: A test of cumulative disadvantage theory. Soc Sci Res. 2008;37(4):1253–66. 19227701 10.1016/j.ssresearch.2008.05.007

[20] Hansen T, Slagsvold B, Veenstra M. Educational inequalities in late-life depression across Europe: results from the generations and gender survey. Eur J Ageing. 2017;14:407–18. 29180946 10.1007/s10433-017-0421-8 PMC5684038

[21] Liu C, Ma Y, Hofman A, Waziry R, Koton S, Pike JR, Windham BG, Power MC, Sharrett AR, Gottesman RF. Educational attainment and dementia: mediation by Mid-Life vascular risk factors. Annals of neurology; 2023. 10.1002/ana.26647 PMC12488220 36966451

[22] Deckers K, Cadar D, van Boxtel MP, Verhey FR, Steptoe A, Köhler S. Modifiable risk factors explain socioeconomic inequalities in dementia risk: evidence from a population-based prospective cohort study. J Alzheimers Dis. 2019;71(2):549–57. 31424404 10.3233/JAD-190541 PMC6839472

[23] Nakahori N, Sekine M, Yamada M, Tatsuse T, Kido H, Suzuki M. A pathway from low socioeconomic status to dementia in Japan: results from the Toyama dementia survey. BMC Geriatr. 2018;18:1–10. 29703157 10.1186/s12877-018-0791-6 PMC5923187

[24] Letellier N, Ilango SD, Mortamais M, Tzourio C, Gabelle A, Empana J-P, Samieri C, Berr C, Benmarhnia T. Socioeconomic inequalities in dementia risk among a French population-based cohort: quantifying the role of cardiovascular health and vascular events. Eur J Epidemiol. 2021;36:1015–23. 34308532 10.1007/s10654-021-00788-8 PMC8542549

[25] Hyun J, Hall CB, Katz MJ, Derby CA, Lipnicki DM, Crawford JD, Guaita A, Vaccaro R, Davin A, Kim KW. Education, occupational complexity, and incident dementia: a COSMIC collaborative cohort study. J Alzheimers Dis. 2022;85(1):179–96. 34776437 10.3233/JAD-210627 PMC8748312

[26] Anderson T. Carol Brayne: a life-course approach to prevent dementia. World Health Organ Bull World Health Organ. 2018;96(3):153–4. 29531413 10.2471/BLT.18.030318 PMC5840636

[27] Bell R, Marmot M. Life course approach to Understanding inequalities in health in later life. Oxf Textbook Geriatric Med 2017:69–76.

[28] Cadar D. A life course approach to dementia prevention. J Aging Geriatric Med 2017, 1(2).

[29] Fratiglioni L, Viitanen M, von Strauss E, Tontodonati V, Herlitz A, Winblad B. Very old women at highest risk of dementia and Alzheimer’s disease: incidence data from the kungsholmen project, Stockholm. Neurology. 1997;48(1):132–8. 9008508 10.1212/wnl.48.1.132

[30] Andersen K, Launer LJ, Dewey ME, Letenneur L, Ott A, Copeland J, Dartigues J-F, Kragh–Sorensen P, Baldereschi M, Brayne C. Gender differences in the incidence of AD and vascular dementia: the EURODEM studies. Neurology. 1999;53(9):1992–1992. 10599770 10.1212/wnl.53.9.1992

[31] Beam CR, Kaneshiro C, Jang JY, Reynolds CA, Pedersen NL, Gatz M. Differences between women and men in incidence rates of dementia and Alzheimer’s disease. J Alzheimers Dis. 2018;64(4):1077–83. 30010124 10.3233/JAD-180141 PMC6226313

[32] Gong J, Harris K, Lipnicki DM, Castro-Costa E, Lima‐Costa MF, Diniz BS, Xiao S, Lipton RB, Katz MJ, Wang C. Sex differences in dementia risk and risk factors: Individual‐participant data analysis using 21 cohorts across six continents from the COSMIC consortium. Alzheimer’s Dement. 2023;19(8):3365–78. 36790027 10.1002/alz.12962 PMC10955774

[33] Kritsotakis G, Psarrou M, Vassilaki M, Androulaki Z, Philalithis AE. Gender differences in the prevalence and clustering of multiple health risk behaviours in young adults. J Adv Nurs. 2016;72(9):2098–113. 27102085 10.1111/jan.12981

[34] Chang SH, Chang YY, Wu LY. Gender differences in lifestyle and risk factors of metabolic syndrome: do women have better health habits than men? J Clin Nurs. 2019;28(11–12):2225–34. 30786102 10.1111/jocn.14824

[35] Dash SR, Hoare E, Varsamis P, Jennings GL, Kingwell BA. Sex-specific lifestyle and biomedical risk factors for chronic disease among early-middle, middle and older aged Australian adults. Int J Environ Res Public Health. 2019;16(2):224. 30650533 10.3390/ijerph16020224 PMC6352175

[36] Krokstad S, Langhammer A, Hveem K, Holmen T, Midthjell K, Stene T, Bratberg G, Heggland J, Holmen J. Cohort profile: the HUNT study, Norway. Int J Epidemiol. 2013;42(4):968–77. 22879362 10.1093/ije/dys095

[37] Åsvold BO, Langhammer A, Rehn TA, Kjelvik G, Grøntvedt TV, Sørgjerd EP, Fenstad JS, Heggland J, Holmen O, Stuifbergen MC. Cohort profile update: the HUNT study, Norway. Int J Epidemiol. 2023;52(1):e80–91. 35578897 10.1093/ije/dyac095 PMC9908054

[38] Then FS, Luck T, Angermeyer MC, Riedel-Heller SG. Education as protector against dementia, but what exactly do we mean by education? Age Ageing. 2016;45(4):523–8. 27055879 10.1093/ageing/afw049

[39] Maccora J, Peters R, Anstey KJ. What does (low) education mean in terms of dementia risk? A systematic review and meta-analysis highlighting inconsistency in measuring and operationalising education. SSM-population Health. 2020;12:100654. 33313373 10.1016/j.ssmph.2020.100654 PMC7721642

[40] Livingston G, Huntley J, Liu KY, Costafreda SG, Selbæk G, Alladi S, Ames D, Banerjee S, Burns A, Brayne C. Dementia prevention, intervention, and care: 2024 report of the lancet standing commission. Lancet. 2024;404(10452):572–628. 39096926 10.1016/S0140-6736(24)01296-0

[41] Bratsberg B, Rogeberg O, Skirbekk V. Technology-induced job loss risk, disability and all-cause mortality in Norway. Occup Environ Med. 2022;79(1):32–7. 34561277 10.1136/oemed-2021-107598 PMC8685638

[42] Yu Q, Li B. Mma: an R package for mediation analysis with multiple mediators. J Open Res Softw 2017, 5(1).

[43] VanderWeele T. Explanation in causal inference: methods for mediation and interaction. Oxford University Press; 2015.

[44] James BD, Bennett DA. Smoking in midlife and dementia in old age: risk across the life course. Arch Neurol. 2011;68(3):365–8. 21403022 10.1001/archneurol.2011.25

[45] Lloyd-Hazlegreaves P, Hayes L, Pearce M. Associations between physical inactivity and dementia prevalence: ecological study using global data. Public Health. 2023;225:299–304. 37956642 10.1016/j.puhe.2023.10.011

[46] Feter N, Mielke GI, Leite JS, Brown WJ, Coombes JS, Rombaldi AJ. Physical activity in later life and risk of dementia: findings from a population-based cohort study. Exp Gerontol. 2021;143:111145. 33189834 10.1016/j.exger.2020.111145

[47] Tekkel M, Ringmets I, Pürjer M-L, Pärna K. Educational differences in cigarette smoking among adult population in Estonia, 1990–2010: does the trend fit the model of tobacco epidemic? 2014. 10.1186/1471-2458-14-709 PMC4226950 25012070

[48] Ruokolainen O, Heloma A, Jousilahti P, Lahti J, Pentala-Nikulainen O, Rahkonen O, Puska P. Thirty-eight-year trends of educational differences in smoking in Finland. Int J Public Health. 2019;64:853–60. 30906956 10.1007/s00038-019-01228-x PMC6614163

[49] Hilal S, Brayne C. Epidemiologic trends, social determinants, and brain health: the role of life course inequalities. Stroke. 2022;53(2):437–43. 35000426 10.1161/STROKEAHA.121.032609

[50] Zhao E, Crimmins EM. Mortality and morbidity in ageing men: biology, lifestyle and environment. Reviews Endocr Metabolic Disorders. 2022;23(6):1285–304. 10.1007/s11154-022-09737-6 PMC9748037 35697963

[51] Crimmins EM, Shim H, Zhang YS, Kim JK. Differences between men and women in mortality and the health dimensions of the morbidity process. Clin Chem. 2019;65(1):135–45. 30478135 10.1373/clinchem.2018.288332 PMC6345642

[52] Daniel RM, Cousens S, De Stavola B, Kenward MG, Sterne J. Methods for dealing with time-dependent confounding. Stat Med. 2013;32(9):1584–618. 23208861 10.1002/sim.5686

[53] Cole SR, Frangakis CE. The consistency statement in causal inference: a definition or an assumption? Epidemiology. 2009;20(1):3–5. 19234395 10.1097/EDE.0b013e31818ef366

[54] Westreich D, Cole SR. Invited commentary: positivity in practice. Am J Epidemiol. 2010;171(6):674–7. 20139125 10.1093/aje/kwp436 PMC2877454

[55] Gjøra L, Strand BH, Bergh S, Borza T, Brækhus A, Engedal K, Johannessen A, Kvello-Alme M, Krokstad S, Livingston G. Current and future prevalence estimates of mild cognitive impairment, dementia, and its subtypes in a population-based sample of people 70 years and older in Norway: the HUNT study. J Alzheimers Dis. 2021;79(3):1213–26. 33427745 10.3233/JAD-201275 PMC7990439

[56] Langhammer A, Krokstad S, Romundstad P, Heggland J, Holmen J. The HUNT study: participation is associated with survival and depends on socioeconomic status, diseases and symptoms. BMC Med Res Methodol. 2012;12(1):1–14. 22978749 10.1186/1471-2288-12-143 PMC3512497

[57] Rojas-Saunero LP, Young JG, Didelez V, Ikram MA, Swanson SA. Considering questions before methods in dementia research with competing events and causal goals. Am J Epidemiol 2023:kwad090. 10.1093/aje/kwad090 PMC10403306 37139580

[58] Valeri L, Proust-Lima C, Fan W, Chen JT, Jacqmin-Gadda H. A multistate approach for mediation analysis in the presence of semi-competing risks with application in cancer survival disparities. ArXiv Preprint arXiv:210213252 2021.

[59] Valeri L, Coull BA. Estimating causal contrasts involving intermediate variables in the presence of selection bias. Stat Med. 2016;35(26):4779–93. 27411847 10.1002/sim.7025

