# Supplementary material for: Mediators of educational differences in dementia risk later in life: evidence from the HUNT study
Source: BMC Public Health. 2025 Apr 10;25:1336. doi: 10.1186/s12889-025-22592-9 (PMC11983785; doi:10.1186/s12889-025-22592-9)
Supplement: Supplementary file 1 — Supplementary Material 1 [file 12889_2025_22592_MOESM1_ESM.docx]

Supplementary Table 1: mediating variable, potential covariates/confounding source and definitions

| **Variables** | **Source** | **Variable defination** | | |
| --- | --- | --- | --- | --- |
|  |  | **Potential confounders and covariates** | | |
| Sex | Statistics Norway | Sex was defined as male vs. female | | |
| Birth year |  | Birth year information was obtained from Statistics Norway | | |
| Number of childern |  | The number of children an individual had was defined as having 1 child, 2, children, 3 children and 4+ children | | |
| Hight in centimeters | The HUNT study | Measured in centimeters | | |
| APE4 allele |  | Defined as has (no ApoE4, has one ApoE4 or has 2 ApoE4). | | |
| **Potental mediators** |  | **stages of life** | | |
|  |  | **Early adulthood (20-44 years)** | **Middle adulthood (45-59 years)** | **Late adulthood (60 years and above)** |
| Smoking | The HUNT study (HUNT1-HUNT3) | Defined as “daily smoker” if the participant reported daily smoking at the age of 44 years or below, “smoked daily before” if they had smoked before and “non-smoker” if they reported not have smoked during up to 44 years or before | Smoking was defined as “daily smoker” if the participant reported daily smoking at age of 45-59 years, “smoked daily before” if they had smoked before and “non-smoker” if they reported not have smoked at age of at age of 45-59 years | Smoking was defined as “daily smoker” if the participant reported daily smoking at age of 60 years or above, “smoked daily before” if they had smoked before and “non-smoker” if they reported not have smoked at age of at age of 60 years or above |
| Physical inactivity |  | Physical inactivity (yes vs. no) was defined as “yes” if participants did not follow the national recommendations for physical activity (30 minutes five times per week), assessed based on self-reported frequency, duration, and intensity of weekly physical activity at the age of 44 years or below and “no” if they did. | Physical inactivity (yes vs. no) was defined as “yes” if participants did not follow the national recommendations for physical activity (30 minutes five times per week), assessed based on self-reported frequency, duration, and intensity of weekly physical activity at ages of 45-59 years and “no” if they did. | Physical inactivity (yes vs. no) was defined as “yes” if participants did not follow the national recommendations for physical activity (30 minutes five times per week), assessed based on self-reported frequency, duration, and intensity of weekly physical activity at ages of 60 years or above “no” if they did. |
| Obesity |  | Obesity was defined using body mass index and an individual was defined as having “obesity” if the participants had a body mass index ≥ 30 Kg/m^2^ at age of 44 years or below vs “not obese” otherwise | Obesity was defined using body mass index and an individual was defined as having “obesity” if the participants had a body mass index ≥ 30 Kg/m^2^ at age of 45-59 years vs. “Not obese” otherwise | Obesity was defined using body mass index and an individual was defined as having “obesity” if the participants had a body mass index ≥ 30 Kg/m^2^ at age of 60 years or above vs. “not obese” otherwise |
| Hypertension |  | The participants were defined as having “hypertension” if they have systolic blood pressure of ≥140 mm Hg or diastolic blood pressure of ≥ 90 mm Hg and/or used antihypertensive mediations at age of 44 years or below vs. not “having hypertension”. | The participants were defined as having “hypertension” if they have systolic blood pressure of ≥140 mm Hg or diastolic blood pressure of ≥ 90 mm Hg and/or used antihypertensive mediations at age of 45-59 years vs. not “having hypertension” otherwise. | The participants were defined as having “hypertension” if they have systolic blood pressure of ≥140 mm Hg or diastolic blood pressure of ≥ 90 mm Hg and/or used antihypertensive mediations at age of 60 years or above vs. not “having hypertension” otherwise. |
| Diabetes |  | Diabetes (yes vs no) was defined as ‘’Yes’’ if they had fasting blood sugar level ≥7mmol/liters and/or reporting having diabetes at age of 44 years or below vs ‘’No’’ if they had fasting blood sugar <7 mmol/liters and/or reported they had no diabetes at age of 44 years or below | Diabetes (yes vs no) was defined as ‘’Yes’’ if they had fasting blood sugar level ≥7mmol/liters and/or reporting having diabetes at age of 45-59 years vs. ‘No’’ if they had fasting blood sugar <7 mmol/liters and/or reported they had no diabetes at age of 45-59 years. | Diabetes (yes vs no) was defined as ‘’Yes’’ if they had fasting blood sugar level ≥7mmol/liters and/or reporting having diabetes at age of 60years or above vs. ‘No’’ if they had fasting blood sugar < 7 mmol/liters and/or reported they had no diabetes at ages of 60years or above. |
| Hearing impairment |  | Hearing impairment (yes vs no) was defined as “yes” if participants reported mild to severe hearing impairment at age of 44 years or below vs. “no” if they did not. | Hearing impairment (yes vs no) was defined as “yes” if participants reported mild to severe hearing impairment at age of 45-59 years vs. “no” if they did not. | Hearing impairment (yes vs no) was defined as “yes” if participants reported mild to severe hearing impairment at age of 60 years or above vs. “no” if they did not. |
| Depression and anxiety symptoms |  | Depression and anxiety symptoms were assessed using the Hospital Anxiety Depression Scale (HADS) score at age of 44 years or below and was used as a continuous variable. | Depression and anxiety symptoms were assessed using the Hospital Anxiety Depression Scale (HADS) score at age of 45-59 years and was used as a continuous variable. | Depression and anxiety symptoms were assessed using the Hospital Anxiety Depression Scale (HADS) score at age of 60 years and above was used as a continuous variable. |
| Low density lipoprotein cholesterol |  | Using the information reported serum cholesterol level, serum high-density lipoprotein cholesterol and serum triglycerides low density lipoprotein (LDL) was calculated using Sampson equation: LDL-C=TC/0.948−HDL-C/0.971−(TG/8.56+TG × non-HDL-C/2140 −TG^2^/16100) − 9.44(1). Then, continuous measures of LDL level at the ages of 44 years or below was defined. | Using the information reported serum cholesterol level, serum high-density lipoprotein cholesterol and serum triglycerides low density lipoprotein (LDL)was calculated using Sampson equation: LDL-C=TC/0.948−HDL-C/0.971−(TG/8.56+TG × non-HDL-C/2140 −TG^2^/16100) − 9.44(1). Then, continuous measures of LDL level at the ages of 45-59 years were defined. | Using the information reported serum cholesterol level, serum high-density lipoprotein cholesterol and serum triglycerides low density lipoprotein (LDL)was calculated using Sampson equation: LDL-C=TC/0.948−HDL-C/0.971−(TG/8.56+TG × non-HDL-C/2140 −TG^2^/16100) − 9.44(1). Then, continuous measures of LDL level at the ages of 60 years or above was defined. |
| Cardiovascular diseases |  | ’Yes’’ if they had myocardial infarction, cerebral hemorrhage at age of 44 years or below vs ‘’no’’ if they did not | ’Yes’’ if they had myocardial infarction, cerebral hemorrhage at age of 45-59 years vs ‘’no’’ if they did not | ’Yes’’ if they had myocardial infarction, cerebral hemorrhage at age of 60 years or above vs ‘’no’’ if they did not |
| Routine task intensity score (RTI) | Statistics Norway | RTI score during early adulthood (age 44 years or below) was defined based on quartiles using standardized scores (1st quartile, 2nd quartile, 3rd quartile, 4th quartile. An additional group, 'Missing or not working,' was generated for those who had no RTI-related information or no working status information,), at the age of 44 years or below. | RTI score during middle adulthood (age 45-59 years) was defined based on quartiles using standardized scores (1st quartile, 2nd quartile, 3rd quartile, 4th quartile. An additional group, 'Missing or not working,' was generated for those who had no work-related information or no working status information,), at the age of 45-59 years. |  |

1. Sampson M, Ling C, Sun Q, Harb R, Ashmaig M, Warnick R, Sethi A, Fleming JK, Otvos JD, Meeusen JW, Delaney SR. A new equation for calculation of low-density lipoprotein cholesterol in patients with normolipidemia and/or hypertriglyceridemia. JAMA cardiology. 2020 May 1;5(5):540-8.
