# Supplementary material for: Mediators of educational differences in dementia risk later in life: evidence from the HUNT study
Source: BMC Public Health. 2025 Apr 10;25:1336. doi: 10.1186/s12889-025-22592-9 (PMC11983785; doi:10.1186/s12889-025-22592-9)
Supplement: Supplementary file 2 — Supplementary Material 2 [file 12889_2025_22592_MOESM2_ESM.pdf]

Mediators of educational differences in dementia risk later in life

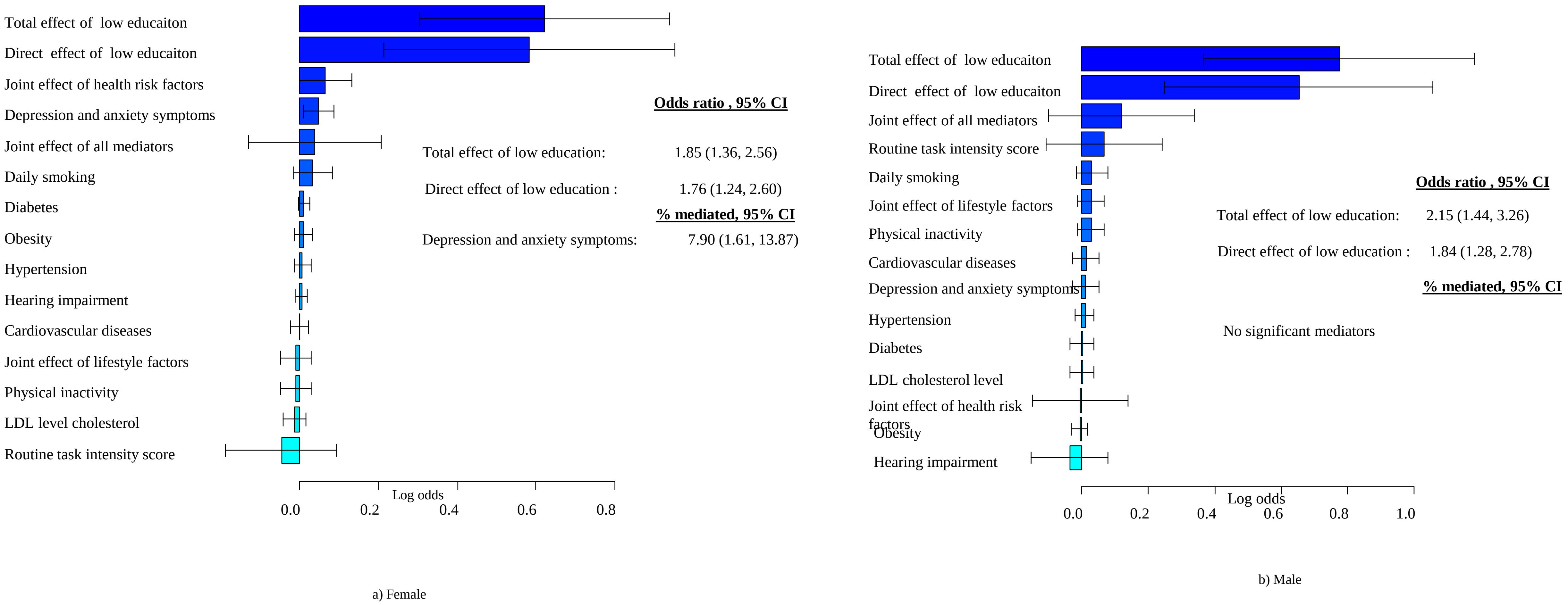

Outcome:dementia (yes vs no); expsoure: educational status (high vs low); lifestyle mediators: smoking (non smoker, daily smoked before, daily smoker), physical inactivity (yes vs no); health risk factor related mediators: hypertension (yes vs no), cardiovascular diseases (yes vs no), obesity (yes vs no), diabetes (yes vs no), hearing impairment (yes vs no); routine task intensity score (defined based on (1st quartile, 2nd quartile, 3rd quartile, 4th quartile and missing or not working)

Supplementary Figure 1: Early adulthood mediators of educational differences in dementia risk in later life, stratified by sex, adjusted for APOE4, height, and birth year.

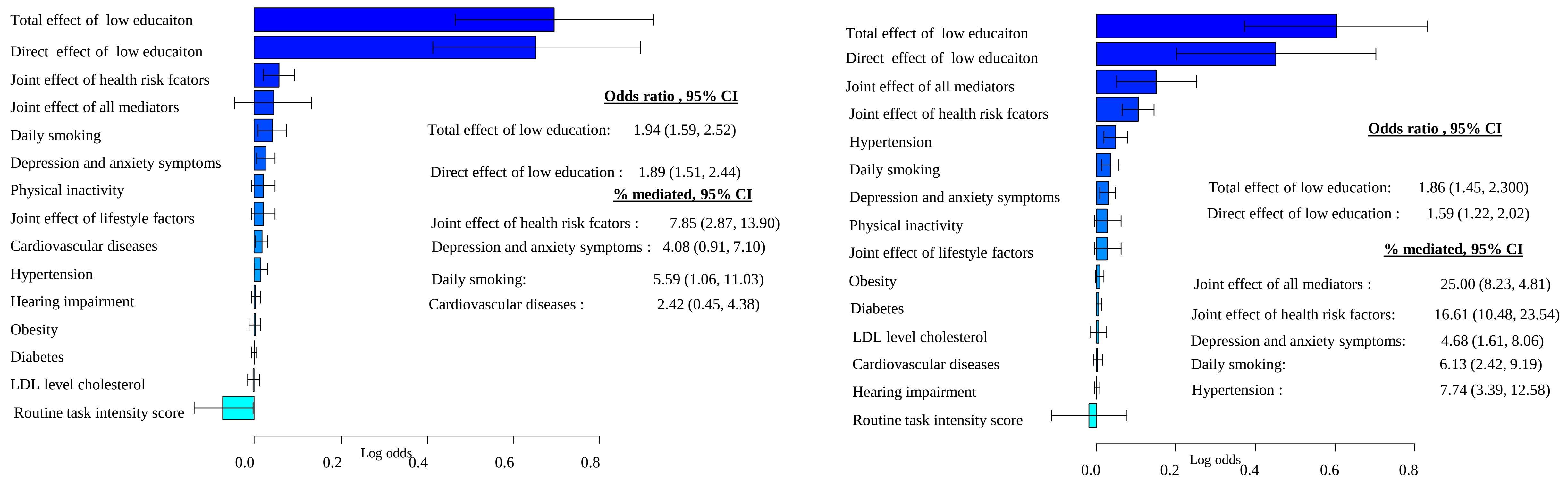

a) Female

b) Male

Outcome:dementia (yes vs no); expsoure: educational status (high vs low); lifestyle mediators: smoking (non smoker, daily smoked before, daily smoker), physical inactivity (yes vs no); health risk factor related mediators: hypertension (yes vs no), cardiovascular diseases (yes vs no), obesity (yes vs no), diabetes (yes vs no), hearing impairment (yes vs no); routine task intensity score (defined based on (1st quartile, 2nd quartile, 3rd quartile, 4th quartile and missing or not working)

Supplementary Figure 2: Middle adulthood mediators of educational differences in dementia risk in later life, stratified by sex, adjusted for APOE4, height, and birth year.

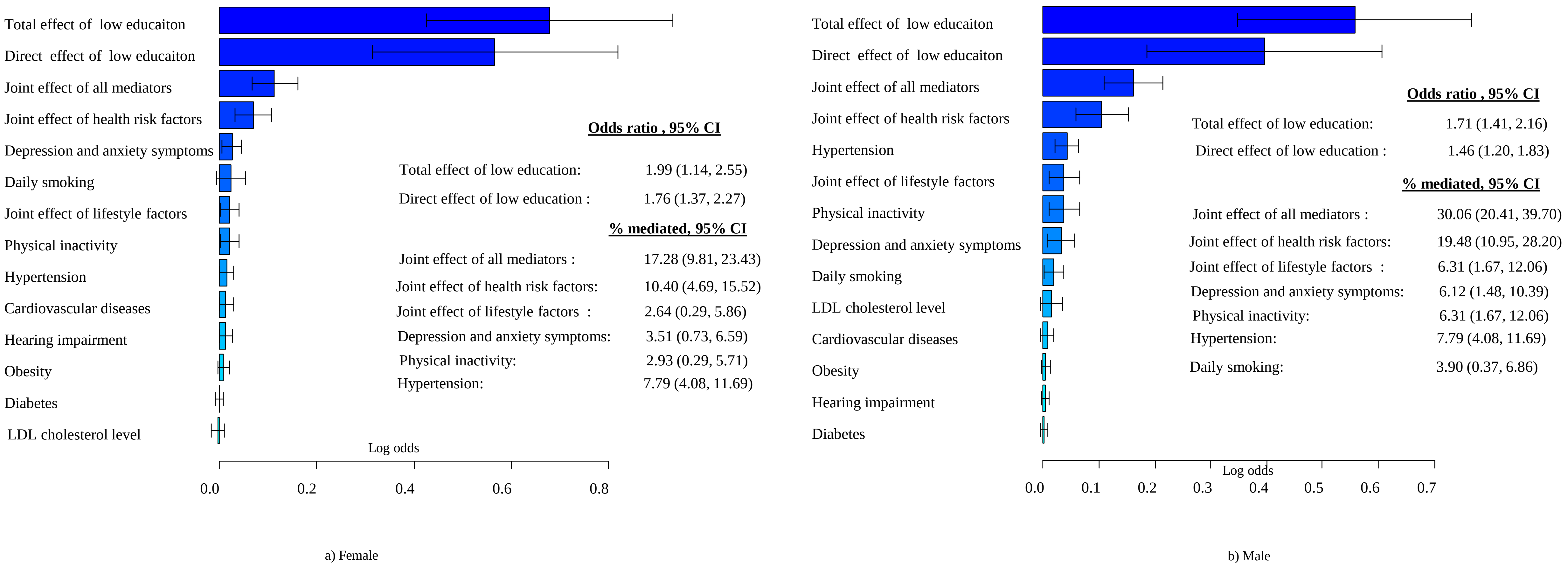

Outcome:dementia (yes vs no); expsoure: educational status (high vs low); lifestyle mediators: smoking (non smoker, daily smoked before, daily smoker), physical inactivity (yes vs no); health risk factor related mediators: hypertension (yes vs no), cardiovascular diseases (yes vs no), obesity (yes vs no), diabetes (yes vs no), hearing impairment (yes vs no

Supplementary Figure 3: Late adulthood mediators of educational differences in dementia risk in later life, stratified by sex, adjusted for APOE4, height, and birth year.

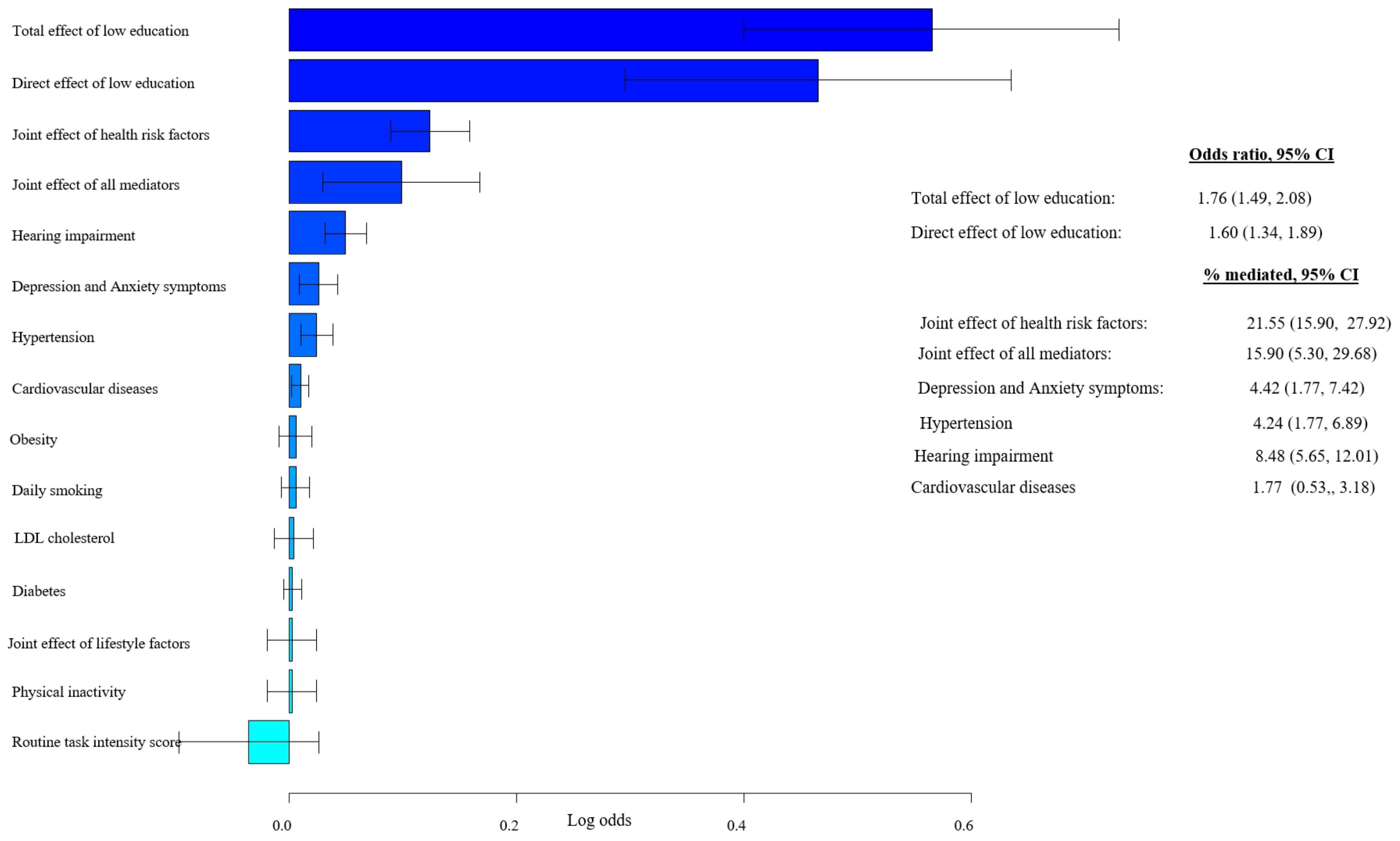

Supplementary Figures 4: Supplementary Figure 4: Mediators of educational differences in dementia risk in later life across all life stages, adjusted for sex, APOE4, height, and birth year.
